# Real-world experience comparing two common left atrial appendage closure devices

**DOI:** 10.1186/s12872-018-0899-9

**Published:** 2018-08-20

**Authors:** Christian Fastner, Lea Hoffmann, Mohamed Aboukoura, Michael Behnes, Siegfried Lang, Martin Borggrefe, Ibrahim Akin, Christoph A. Nienaber

**Affiliations:** 1 First Department of Medicine, University Medical Centre Mannheim (UMM), Faculty of Medicine Mannheim, University of Heidelberg, European Centre for AngioScience (ECAS), and DZHK (German Centre for Cardiovascular Research) partner site Heidelberg/Mannheim, Theodor-Kutzer-Ufer 1-3, 68167 Mannheim, Germany; 2 Department of Cardiology, University Hospital Rostock, Rostock, Germany; 3 Royal Brompton Hospital, London, United Kingdom and National Heart and Lung Institute, Imperial College London, London, UK

**Keywords:** Atrial fibrillation, Left atrial appendage, Left atrial appendage closure device, Outcome, Comparison

## Abstract

**Background:**

The interventional left atrial appendage closure (LAAC) is a guideline-conform alternative to oral anticoagulation (OAC) in non-valvular atrial fibrillation patients with OAC ineligibility. It was aimed to directly compare two contemporary devices in a real-world patient population.

**Methods:**

LAAC was conducted in two centres between 2010 and 2014 as well as between 2014 and 2017, respectively, in a standard fashion based on the specific manufacturer’s recommendations. Baseline characteristics, procedural data and event rates during intra-hospital and 6 months follow-up were registered in a retrospective approach, and analysed in device-related groups.

**Results:**

A total of 189 patients presented for LAAC device implantation. Baseline characteristics were mostly evenly distributed. In 148 patients, a Watchman™ device (Boston Scientific, Natick, MA, USA) was successfully implanted, an Amplatzer™ Amulet™ (St. Jude Medical, St. Paul, MN, USA) in 34 patients (96.1 and 97.1%, respectively; *p* = 1.00). Major access site bleedings were more frequent in the Amplatzer™ Amulet™ group (8.9 versus 1.4%; *p* = 0.046). No intra-hospital thromboembolic event was present. During 6 months follow-up, peri-device leaks > 5 mm and thromboembolic events were uncommon (each *p* = n.s.).

**Conclusions:**

While procedural success was equally high with both contemporary devices, complications during follow-up were rare, and evenly distributed.

## Background

Atrial fibrillation (AF) is the most common cardiac arrhythmia with an age-dependent prevalence from 0.1% among < 55 year olds to 9% in octogenarians [1]. Stroke and systemic embolization are prognostically relevant complications [2]. In patients with an increased risk for thromboembolism under AF, identified by a CHA_2_DS_2_-VASc score ≥ 2 in men and ≥ 3 in women, systemic oral anticoagulation (OAC) is the guideline conform prophylactic treatment [3]. However, an underuse of these substances is observed in daily practice despite the introduction of non-Vitamin K antagonist oral anticoagulants (NOACs) [4–6]. Typical contraindications for long-term OAC are relevant prior bleedings with a tendency to recidivity, a high predisposition for major bleeding events, other adverse drug reactions, the need for dialysis or the individual patient’s refusal [7]. Moreover, some patients suffer from thromboembolic strokes despite adequate OAC [8].

Within the last decade, the interventional left atrial appendage closure (LAAC) was implemented as a prophylactic alternative in all these above-mentioned cases. Currently, it is recommended by the European guidelines on atrial fibrillation (class IIb) in all patients with contraindications to long-term OAC [3]. This locoregional technique rests on the observation that > 90% of all emboli related to non-valvular AF originate from the left atrial appendage (LAA) [9]. The LAAC with the WATCHMAN™ device (Boston Scientific, Natick, MA, USA) was proven to be non-inferior to long-term OAC for the combined efficacy outcome of stroke, systemic embolization and cardiovascular death in a randomized controlled trial (RCT) [10]. After 3.8 years, the interventional approach was superior to OAC with respect to the combined study endpoint [11]. While the patients in the RCT were anticoagulated for at least 45 days after the procedure, dual antiplatelet agents were shown to be an effective, and safe antithrombotic alternative in all those patients with an absolute contraindication for OAC [12, 13].

Meanwhile, two large registries confirmed efficacy and safety for both common devices, i.e., the WATCHMAN™ device and the AMPLATZER™ Cardiac Plug (St. Jude Medical, St. Paul, MN, USA), in a real-world patient collective [14, 15]. Particularly, the periinterventional complication rates revealed to be much lower than in the initial RCT [14, 16, 17]. Thus, both devices have proven their practical applicability. However, the side-by-side comparison of the two contemporary devices is limited to few data [18]. Based on the assumption that not all the information derived from studies on one device is one-to-one applicable to the other, this study aimed to compare the WATCHMAN™ device to the second-generation AMPLATZER™ Amulet™ regarding patient characteristics, procedural success and complications during follow-up.

## Methods

### Enrollment

This study is based on retrospective observational registry data from two German centres. Centre 1 (University Hospital Rostock, Rostock, Germany) performed LAAC with the WATCHMAN™ device, centre 2 (University Medical Centre Mannheim, Mannheim, Germany) with the WATCHMAN™ device and the AMPLATZER™ Amulet™. Enrollment period lasted from 2010 to 2014 in centre 1 and from 2014 to 2017 in centre 2. Both centres aimed to include consecutively all LAAC cases to avoid a recruitment bias. Patient characteristics, implantation details including complications and follow-up data was extracted from the original medical documents. The methods were carried out in accordance with the relevant local guidelines and regulations. All protocols were approved by the medical ethics committee of the Faculty of Medicine, University of Rostock, Germany, and the medical ethics committee II of the Faculty of Medicine Mannheim, University of Heidelberg, Germany. Due to the retrospective data acquisition, written informed consent concerning the study was not obtained but all patients consented in the conduction of the procedure beforehand.

### Procedure and intra-hospital follow-up

The operators’ experience and the conduction of the implantation procedure were comparable in both centres. Specific manufacturer’s recommendations were considered. The procedure was performed under conscious sedation, and guided by fluoroscopy, angiography and transoesophageal echocardiography (TOE) in all cases. Device selection in centre 2 was left to the operator’s discretion based on preprocedural TOE measurements (orifice and landing zone’s diameter, LAA depth, morphology). After device releasing and sheath removal the venous access site was sealed at the discretion of the operator (Z-suture, Perclose ProGlide™ (Abbott, Redwood City, CA, USA)), an arterial access (femoral 5 French sheath), which had been established in some cases, was sealed with manual compression or Angio-Seal™ vascular closure device (Terumo, Shibuya, Japan). Following the procedure, stable device position and potential peri-device leaks were identified by TOE. A thorough clinical examination served to identify neurological or access site complications. In centre 1, postprocedural antithrombotic regimen was individualised, while all patients in centre 2 received acetylsalicylic acid (ASA) lifelong and additional clopidogrel for 6 months.

### Mid-term follow-up

In the context of the clinical routine, patients presented 6 months after the procedure for a follow-up visit. A TOE as well as a clinical re-examination were conducted during this visit.

### Outcome measures

Successful device implantation was defined in the absence of a relevant peri-device leak, i.e., > 5 mm. A bleeding was categorized as “major bleeding” when the event could be attributed to Bleeding Academic Research Consortium (BARC) definition ≥ type 3. The primary efficacy outcome measure was the absence of stroke and systemic embolization during follow-up, a secondary efficacy outcome measure was successful device deployment. Safety was assessed by the absence of any complication related to the intervention or the postinterventional antithrombotic regimen. Events which could not be traced back to the intervention or the related medical therapy were registered as adverse events.

### Statistics

Statistical analyses were performed with SPSS Statistics (IBM, Armonk, NY). Continuous data are presented as means with standard deviation, categorical data as total numbers with group-related percentages. Between the device groups, categorical variables were compared using the chi-squared test or the Fisher’s exact test for rare events. The unpaired t-test with Welch correction for unequal variances was applied to compare continuous variables. The statistics were based on the available cases per item. *P* values < 0.05 (two-tailed) were considered statistically significant.

## Results

### Baseline characteristics

Baseline demographic and clinical characteristics of the study population including risk stratification according to the CHA_2_DS_2_-VASc and the HAS-BLED scores are displayed in Table 1. A total of 189 patient cases could be included in this registry. Ninety-seven patients were indicated for LAAC in centre 1 and 92 in centre 2. The population presented with a mean CHA_2_DS_2_-VASc score of 4.4 ± 1.5 (*p* = 0.06 in comparison of both devices) and a mean HAS-BLED score of 3.6 ± 1.1 (*p* = 0.12). 76.7% of patients had a HAS-BLED score ≥ 3 points. In general, baseline characteristics were statistically evenly distributed between the two device groups, except for relevant prior bleeding events, which were significantly more common in patients that received an Amplatzer™ Amulet™ (*p* = 0.008). Consequently, contraindication for long-term OAC was significantly more often defined by a prior bleeding event in the Amplatzer™ Amulet™ group (*p* = 0.032). Irrespective of the device group, a prior bleeding event was the most common indication for the intervention (64.0% of the overall population).

**Table 1:** Baseline characteristics

	Watchman™ (*n* = 154)	Amplatzer™ Amulet™ (*n* = 35)	*p* value^*^	OR (95% CI)
Male, n (%)	105 (68.2)	22 (62.9)	0.55	1.27 (0.58–2.73)
Age [years], mean ± SD	75.2 ± 2.8	77.1 ± 9.7	0.27	–
CHA_2_DS_2_-VASc score, mean ± SD	4.5 ± 0.1	4.0 ± 1.4	0.06	–
HAS-BLED score, mean ± SD	3.6 ± 0.2	3.7 ± 1.0	0.12	–
HAS-BLED score ≥ 3, *n* (%)	127 (82.5)	33 (94.3)	0.12	3.5 (0.79–15.52)
Type of AF, each *n* (%)				
Paroxysmal	61 (39.6)	17 (48.6)	0.35	0.69 (0.31–1.55)
Persistent	41 (26.6)	4 (11.4)	0.08	3.72 (1.17–13.07)
Permanent	45 (29.2)	14 (40.0)	0.23	0.62 (0.27–1.42)
Unknown	7 (4.5)	0 (0.0)	0.35	–
Congestive heart failure, *n* (%)	47 (30.5)	8 (22.9)	0.42	0.67 (0.28–1.60)
Arterial hypertension, *n* (%)	148 (96.1)	34 (97.1)	1.00	1.38 (0.16–11.84)
Diabetes mellitus, *n* (%)	52 (33.8)	14 (40.0)	0.56	1.31 (0.61–2.79)
Prior cerebrovascular event, each *n* (%)	44 (28.6)	8 (22.9)	0.54	0.74 (0.31–1.76)
Vascular disease, *n* (%)	98 (63.6)	19 (54.3)	0.34	0.68 (0.32–1.43)
Chronic kidney disease, *n* (%)	54 (35.1)	12 (34.3)	1.00	0.97 (0.44–2.10)
Chronic liver disease, *n* (%)	10 (6.5)	4 (11.4)	0.30	1.86 (0.54–6.32)
Prior bleeding, *n* (%)	100 (64.9)	31 (88.6)	**0.008**	4.19 (1.40–12.49)
Bleeding localization, each *n* (%)				
Intracranial	28 (18.2)	6 (17.1)	1.00	1.07 (0.37–3.20)
Gastrointestinal	57 (37.0)	20 (57.1)	**0.036**	0.44 (0.73–1.00)
Muscle	3 (1.9)	0 (0.0)	1.00	–
Skin/mucosal	4 (2.6)	3 (8.6)	0.12	0.28 (0.05–1.70)
Other/unknown	8 (5.2)	2 (5.7)	1.00	0.90 (0.16–6.49)
Indication for LAAC, each *n* (%)				
Prior bleeding	93 (60.4)	28 (80.0)	**0.032**	0.38 (0.14–0.91)
Drug intolerance	25 (16.2)	1 (2.9)	0.05	6.59 (1.12–69.94)
LAA thrombus despite OAC	4 (2.6)	1 (2.9)	1.00	0.91 (0.14–11.39)
Thromboembolic event despite OAC	3 (1.9)	2 (5.7)	0.23	0.33 (0.06–1.92)
Patient’s preference	9 (5.8)	0 (0.0)	0.21	–
Other reason	20 (13.0)	3 (8.6)	0.58	1.59 (0.47–5.32)

*AF* Atrial fibrillation, *CI* Confidence interval, *IQR* Interquartile range, *LAA(C)* Left atrial appendage (closure), *OAC* Oral anticoagulation, *OR* Odds ratio, *SD* Standard deviation

*Fisher’s exact or unpaired t-test for the comparison of both groups, *p* < 0.05 indicates statistical significance

### Procedural data

Technical success – defined as stable device anchorage and absence of a peri-device leak > 5 mm at the end of the procedure – could be achieved in 96.3% of all patients (*p* = n.s. between the device groups; Table 2). Four patients of the Watchman™ group were implanted in a second procedure, as they revealed a LAA thrombus during the initial intervention which could be successfully resolved by short-term OAC. Out of the 6 patients without implantation success, 5 had a wide and tub-shaped LAA orifice and neck region which was not providing conditions for adequate device anchorage. In another patient, the LAA thrombus could not be resolved despite proper anticoagulation, and the remaining LAA was too small to implant the device. One implantation failure in the Amplatzer™ Amulet™ group was due to a circulatory collapse and subsequent death in a patient with a highly reduced left ventricular (LV) function.

**Table 2:** Procedural data and intra-hospital outcome

	Watchman™ (*n* = 154)	Amplatzer™ Amulet™ (*n* = 35)	*p* value*	OR (95% CI)
Successful implantation, *n* (%)	148 (96.1)	34 (97.1)	1.00	1.38 (0.16–11.84)
Intraprocedural device dislodgement, *n* (%)	2 (1.3)	0 (0.0)	1.00	0.86 (0.04–18.31)
	Watchman™ (successful *n* = 148)	Amplatzer™ Amulet™ (successful *n* = 34)	*p* value*	OR (95% CI)
Peri-device leak < 5 mm, *n* (%)	2 (1.4)	0 (0.0)	1.00	0.85 (0.03–18.11)
Access site bleeding, each *n* (%)	12 (8.1)	6 (17.6)	0.11	0.41 (0.12–1.36)
Minor bleeding	6 (4.1)	2 (5.9)	0.64	1.48 (0.28–7.68)
Major bleeding	2 (1.4)	3 (8.9)	**0.046**	7.07 (1.13–44.09)
Pseudoaneurysm	4 (2.7)	1 (3.0)	1.00	1.09 (0.11–10.01)
Pericardial effusion	12 (8.1)	2 (5.9)	1.00	1.41 (0.27–9.64)
Without hemodynamic impact	7 (4.7)	2 (5.9)	0.68	0.79 (0.14–5.83)
With hemodynamic impact	3 (2.0)	0 (0.0)	1.00	–
Tamponade	2 (1.4)	0 (0.0)	1.00	–
Intra-hospital stroke, *n* (%)	0 (0.0)	0 (0.0)	1.00	–
Postprocedural device dislodgement, *n* (%)	1 (0.7)	0 (0.0)	1.00	1.43 (0.06–35.77)
Intra-hospital death, *n* (%)	1 (0.7)	1 (2.9)	0.34	4.46 (0.27–73.12)
Postprocedural antithrombotic therapy in the first 6 months, each *n* (%)				
DAPT	140 (94.6)	34 (100.0)	0.36	–
OAC plus clopidogrel	2 (1.4)	0 (0.0)	1.00	–
45 days LMWH plus one antiplatelet agent, DAPT afterwards	6 (4.1)	0 (0.0)	0.60	–

*ASA* Acetylsalicylic acid, *CI* Confidence interval, *DAPT* Dual antiplatelet therapy, *LMWH* Low molecular weight heparin, *OAC* Oral anticoagulation, *OR* Odds ratio

*Fisher’s exact test for the comparison of both groups, *p* < 0.05 indicates statistical significance

Both intraprocedurally dislodged Watchman™ devices were successfully snared in the LA cavity, and retrieved through a stable transseptal electrophysiological sheath [19]. In one case, within the same session, a larger Watchman™ device could successfully be implanted.

While all patients were discontinued with OAC in centre 2 after successful device implantation, 94.6% of the successfully implanted patients received dual antiplatelet therapy (DAPT) with ASA and clopidogrel for 6 months in centre 1 as well. Only a small minority of patients received anticoagulants after successful LAAC. Six patients were prescribed low molecular weight heparins plus an antiplatelet agent for 45 days followed by DAPT with ASA and clopidogrel for half a year. Additional two patients were treated with phenprocoumon and clopidogrel for 6 months. All patients should continue to receive ASA from month 7 onwards for the rest of their lives.

### Postprocedural complications and safety events

Postprocedural major access site bleedings were statistically more common in the Amplatzer™ Amulet™ group (*p* = 0.046; Table 2). All five cases of major bleeding, i.e., bleeding defined by a BARC score ≥ 3, needed blood transfusions, however, none had to be operated. Five pseudoaneurysms were successfully treated by ultrasound-guided compression. Pericardiocentesis was sufficient to resolve 2 pericardial tamponades and 3 hemodynamically relevant pericardial effusions. Nine minor pericardial effusions could be treated conservatively. Heart surgery was needed to retrieve a post-procedurally dislodged Watchman™ device in 1 case.

The above-mentioned peri-procedural death occurred directly after transseptal puncture. Both, air embolism and pericardial tamponade could be ruled out by a thorough analysis of the underlying cause. Rather, deep conscious sedation in connection with a highly-depressed LV function was stated as cause of death. Two more patients died during hospital stay after successful device implantation, however, none of these deaths was linked to the procedure (urosepis and hypokalaemia-induced ventricular fibrillation), and, therefore, termed adverse events. None of the surviving patients developed persistent disability.

### Follow-up

One hundred twelve patients in the Watchman™ group (75.7% of the implanted patients) and 30 patients in the Amplatzer™ Amulet™ group (88.2% of the implanted patients) presented at 6 months’ follow-up visit (Table 3). One hundred two Watchman™ patients (68.9% of the implanted patients) and 21 Amplatzer™ Amulet™ patients (61.8% of the implanted patients) underwent a TOE examination at follow-up. Meanwhile, device dislodgement in the abdominal aorta was incidentally detected by computed tomography conducted for other reasons in 1 additional patient of the Amplatzer™ Amulet™ group (*p* = 1.00 versus the Watchman™ group), and the device could be successfully retrieved in a catheter-based intervention. In all patients that presented for 6 months’ follow-up TOE, the device was detectable in the LAA. Meanwhile, 1 patient of the Watchman™ group with a highly-depressed LV function revealed a LV thrombus 1 months after the implantation which could be resolved by 4 weeks of heparin therapy. The treating physicians decided to prolong the DAPT in a patient whose Watchman™ device was rotated 90° in the LAA, and, therefore, had a peri-device leak > 5 mm to achieve LAA thrombosis nonetheless. Six patients died during follow-up. However, none of these cases could be directly traced to the device implantation nor to the dual antiplatelet therapy. In 1 patient, major bleeding after iatrogenic vascular injury during thoracentesis resulted in haemorrhagic shock and death, however, this was not primarily attributed to the DAPT, but rather to the massive trauma.

**Table 3:** Six months follow-up data

	Watchman™ (clinical follow up: *n* = 112; TOE follow-up: *n* = 102)	Amplatzer™ Amulet™ (clinical follow-up: *n* = 30; TOE follow-up: *n* = 21)	*p* value*	OR (95% CI)
Device detectable in LAA, *n* (%)	102 (100.0)	21 (100.0)	1.00	–
Peri-device leak < 5 mm, *n* (%)	14 (13.7)	2 (9.5)	1.00	0.66 (0.13–3.16)
Peri-device leak > 5 mm, *n* (%)	1 (1.0)	0 (0.0)	1.00	–
Device thrombus, *n* (%)	4 (4.9)	0 (0.0)	0.59	0.51 (0.02–9.82)
Pericardial effusion, *n* (%)	2 (2.0)	0 (0.0)	1.00	–
Minor bleeding, *n* (%)	4 (3.6)	1 (3.3)	1.00	1.07 (0.11–6.24)
Major bleeding, *n* (%)	6 (5.4)	2 (6.7)	0.68	0.79 (0.13–6.04)
Thromboembolic event, *n* (%)	1 (0.9)	0 (0.0)	1.00	1.22 (0.04–30.70)
Death, *n* (%)	4 (3.6)	2 (6.7)	0.61	1.93 (0.33–11.08)

*CI* Confidence interval, *LAA* Left atrial appendage, *OR* Odds ratio, *TOE* Transoesophageal echocardiography

*Fisher’s exact test for the comparison of both groups, *p* < 0.05 indicates statistical significance

### Transoesophageal echocardiographic measurements

Table 4 summarizes the TOE measurements of dimensions at baseline and 6 months’ follow-up visit of the patients from centre 2. The Watchman™ device was compressed to a significantly higher degree than the Amplatzer™ Amulet™ (77 versus 85%, respectively; *p* = 0.015).

**Table 4:** Transoesophageal echocardiographic measurements

	Watchman™	Amplatzer™ Amulet™	*p* value*
Baseline TOE			
LAA morphology, each n (%)			0.14
Windsock	22 (38.6)	11 (31.4)	
Cauliflower	16 (28.1)	10 (28.6)	
Chicken wing	14 (24.6)	5 (14.3)	
Cactus	5 (8.8)	9 (25.7)	
LA diameter, mean ± SD	50.6 ± 1.0	48.7 ± 1.4	0.27
LA surface, mean ± SD	24.1 ± 1.2	23.8 ± 1.2	0.83
LAA depth, mean ± SD	29.5 ± 1.1	29.2 ± 1.6	0.87
LAA orifice diameter, mean ± SD			
45°	19.0 ± 0.6	20.5 ± 0.9	0.16
90°	19.6 ± 0.7	20.4 ± 0.9	0.46
135°	20.7 ± 1.2	21.1 ± 1.0	0.82
LAA landing zone diameter, mean ± SD	18.4 ± 0.8	18.6 ± 0.8	0.87
Follow-up TOE			
LA diameter, mean ± SD	48.5 ± 1.4	45.7 ± 2.8	0.37
LA surface, mean ± SD	21.8 ± 1.0	22.4 ± 1.3	0.71
LAA orifice diameter, mean ± SD			
45°	20.0 ± 2.0	24.4 ± 3.5	0.30
90°	22.7 ± 0.8	23.4 ± 1.1	0.57
135°	20.4 ± 2.3	22.5 ± 4.5	0.73
Device diameter post-implantation / initial device diameter, mean ± SD	0.77 ± 0.03	0.85 ± 0.02	**0.015**

*LA(A)* Left atrial (appendage), *SD* Standard deviation, *TOE* Transoesophageal echocardiography

*Fisher’s exact or unpaired t-test for the comparison of both groups, *p* < 0.05 indicates statistical significance; based on the available data of 92 patients from centre 2 with TOE at baseline and 53 patients from centre 2 with TOE at follow-up visit

## Discussion

This two-centre registry retrospectively analysing a real-world population of non-valvular AF patients that underwent interventional closure of the LAA, confirmed excellent efficacy and safety of the interventional approach in both contemporary devices. Regarding relevant outcome parameters, no significant difference was seen in comparison of both devices.

The study collective was both, at high risk for stroke (mean CHA_2_DS_2_-VASc score 4.4 ± 1.5; *p* = 0.06) and for bleeding events (mean HAS-BLED score 3.6 ± 1.1; *p* = 0.12) which is in line with the European guideline requirements for LAAC [3]. Moreover, the risk profiles were more pronounced than those in the initial approval studies which were conducted in patients eligible for OAC [10, 20], and they certainly reflect the present clinical situation. The baseline characteristics of our collective are in good accordance with those of recently published large registries evaluating outcomes in any one device [14, 16]. Of note, baseline characteristics do not significantly differ between the device groups, facilitating the evaluation of the intervention’s impact on outcome parameters, except for the registered rate of prior bleeding events.

**Fig. 1:**
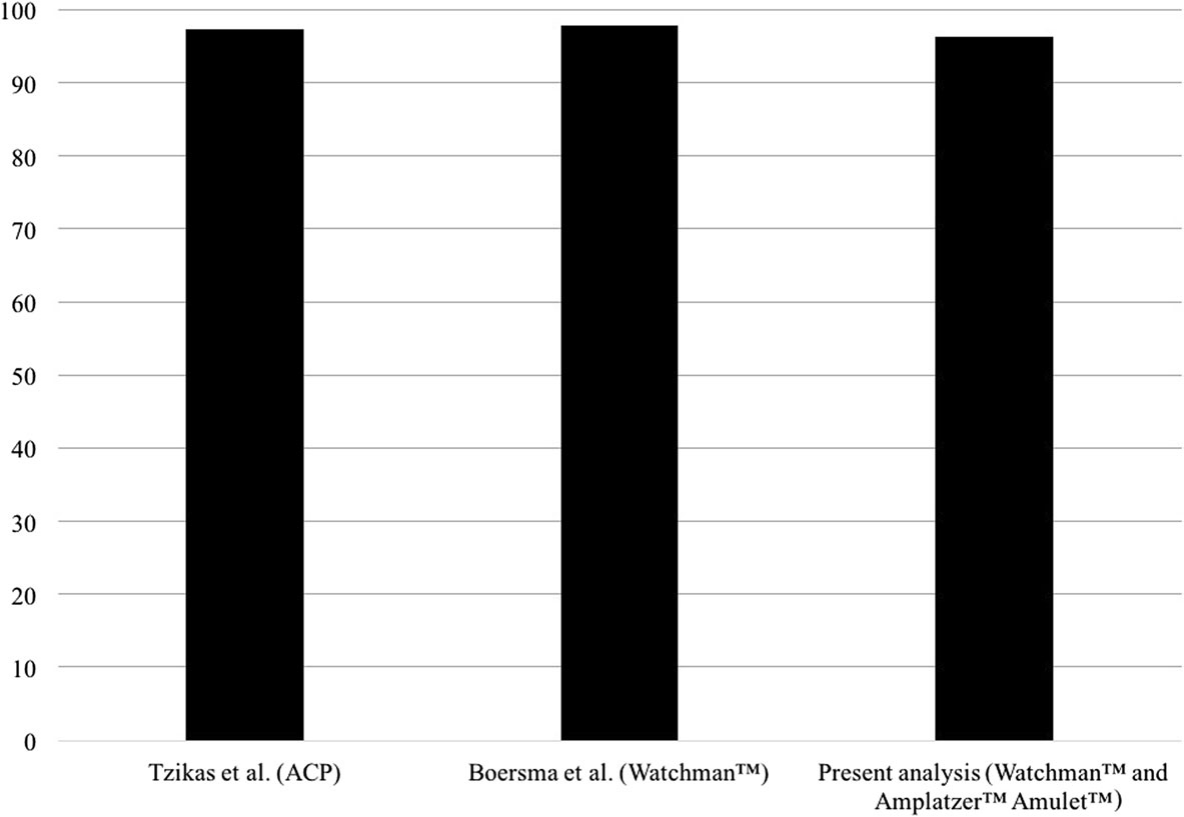
Implantation success (percentage) of relevant recent real-world registries compared to the present analysis; ACP = Amplatzer™ Cardiac Plug

Concerning the secondary efficacy outcome measure, success in device deployment (96.3% for both devices; *p* = n.s.) was comparable high to previously published real-world data (Fig. 1) [14, 16, 18]. Operators in both centres were well trained (≥50 prior implantations each) or were guided by an experienced operator. This is also depicted by the fact, that intra-procedural device dislodgements could be handled within the same intervention without any further harm to the patient. The 4 patients in which procedural success could only be reached after thrombus resolution highlight that boundaries between patients eligible and ineligible for OAC are fluid. Therefore, the long-term ineligibility for OAC might be the guiding principle [3] while short periods of reinitiated anticoagulation may be tolerated by some patients. Besides the classic indication “prior bleeding”, ineligibility was also given due to a LAA thrombus and/or thromboembolic event under adequate OAC [21]. Among the cases with “other” indications, the interventional approach is of special interest to AF patients with end-stage renal failure on dialysis as these patients have a tremendously increased stroke risk while they do not seem to profit from medical prophylaxis [22].

The cut-off set for acceptable peri-device leaks is closely linked to the definition of procedural success. Just like in any relevant previous study since the initial PROTECT-AF trial, both centres chose a cut-off of 5 mm as for smaller peri-device leaks clinical irrelevance is assumed [23]. Any type of peri-device leak was infrequent in both groups (*p* = 1.0) and no device dislodgement was detected during follow-up. This was even though the Watchman™ device appeared significantly more compressed in follow-up TOE (*p* = 0.015). Limited to a 6 months post-intervention period, the LAAC technique’s efficacy, i.e., the primary outcome measure, was demonstrated by only 1 thromboembolic event which occurred in a total of 142 clinically followed-up patients (0.7%; *p* = 1.00). As a deep vein thrombosis was detected being causal for a pulmonary embolism, this case was not to be registered as a cardioembolic event, but, nevertheless, it occurred based on OAC cessation after LAAC. More and more frequently, AF is characterized by a more comprehensive definition than being reduced to an atrial disorder with locoregional thrombogenic potential in the LAA. Indeed, AF seems to be associated with a general state of hypercoagulability [24] which could also be an explanation for the occurrence of the deep vein thrombosis.

By means of safety, the rate of major access site bleedings in the Amplatzer™ Amulet™ group (8.9 versus 1.4% of all implantations; *p* = 0.046) appears surprisingly high compared to 0.8% of major access site bleedings in the registry of Tzikas et al. with the first generation Amplatzer™ device [14]. The Amplatzer™ Amulet™ is not known to increase this rate [25]. It might be speculated, that the number of relevant access site bleedings may be overestimated by the small number of Amulet™ implantations and by the heterogeneous use of vascular closure techniques. However, this finding emphasis the fact that advantages of the interventional approach can only be achieved in the long run while the initial period is determined by complications of the complex procedure [20]. Of note, only one procedure-related death had to be registered, and no periprocedural thromboembolic event occurred.

Within follow-up, 5.6% of patients faced a major bleeding event (*p* = 0.68). All patients received more than one antithrombotic agent during the follow-up period, the majority a DAPT (95.6%; *p* = 0.36). Though being used as an alternative to OAC for ineligible patients in clinical practice, DAPT was shown not to be favourable to warfarin in lowering the bleeding risk [26]. Once again, the intervention’s net benefit should be expected only after several years [11].

### Study limitations

These analyses were based on retrospective observational registry data with the inherent limitations of this study type, e.g., a selection bias. Due to the retrospective character of this registry, conduction of the intervention was not influenced by the study investigators, and based on the operators’ discretion. This individualized decision algorithm might have not insignificantly influenced the outcome measures but surely reflects the clinical practice. The operators could choose between two device types in only one centre, which created an imbalance in the total number of device implantations. Concerning the registration of bleeding events, the reservation must be made, that the grading of bleeding events unlike all other baseline characteristics was not objectively extractable out of the available patient data, but rather was based on the subjective assessment and documentation of the treating physician. Apparently, a bleeding event was more often considered relevant in centre 2. Since in centre 1 only the Watchman™ device was implanted, this circumstance impacted the proportional distribution of prior bleeding events. In addition, a HAS-BLED score > 3 was numerically more frequent in the Amplatzer™ Amulet™ group. Due to an imbalance in the total number of device implantations per group, this numerical variance might not be reflected by a statistical significant difference. That is also why we did not elaborate a regression analysis of outcome parameters on the factor “prior bleeding event”. A follow-up TOE was available in only 69 and 62% of all successfully implanted patients, respectively, and the follow-up period was limited to 6 months, and, therefore, rates of thromboembolism and major bleedings could not be compared to the estimated annual rates from the CHA_2_DS_2_-VASc and the HAS-BLED score. Moreover, the limited sample size might not have insignificantly contributed to the non-detection of thromboembolic events during follow-up as these events are known to be infrequent after the LAAC procedure. However, despite the limitations of this observational registry, it is serving as a data source for a little studied topic.

## Conclusions

Independent from the selected device type, technical success was high, and the interventional closure of the LAA presented with adequate efficacy and safety within 6 months follow-up in a real-world population. While this registry provides first insights into the comparison of different LAA closure devices in clinical practice, larger, whenever possible randomized studies or well-designed prospective registries will have to confirm these results.

## Abbreviations

(N)OAC: (Non-Vitamin K antagonist) oral anticoagulation
AF: Atrial fibrillation
ASA: Acetylsalicylic acid
BARC: Bleeding Academic Research Consortium
CI: Conficence interval
DAPT: Dual antiplatelet therapy
IQR: Interquartile range
LA(A): Left atrial (appendage)
LAAC: Left atrial appendage closure
LMWH: Low molecular weight heparin
LV: Left ventricular
n.s.: Not significant
OR: Odds ratio
RCT: Randomized controlled trial
SD: Standard deviation
TOE: Transoesophageal echocardiography
